# Dosimetric and radiobiological comparison between conventional and hypofractionated breast treatment plans using the Halcyon system

**DOI:** 10.3389/fonc.2023.1259416

**Published:** 2023-09-26

**Authors:** Duong Thanh Tai, Luong Tien Phat, Nguyen Ngoc Anh, Huynh Van Tran Sang, Tran Minh Loc, Nguyen Xuan Hai, Peter A. Sandwall, David Bradley, James C. L. Chow

**Affiliations:** ^1^ Department of Medical Physics, Faculty of Medicine, Nguyen Tat Thanh University, Ho Chi Minh, Vietnam; ^2^ Robarts Research Institute, University of Western Ontario, London, ON, Canada; ^3^ Department of Radiation Oncology, University Medical Shing Mark Hospital, Bien Hoa, Vietnam; ^4^ Faculty of Fundamental Science, PHENIKAA University, Hanoi, Vietnam; ^5^ PHENIKAA Research and Technology Institute (PRATI), A&A Green Phoenix Group JSC, Hanoi, Vietnam; ^6^ Dalat Nuclear Research Institute, Dalat, Vietnam; ^7^ Department of Radiation Oncology, OhioHealth, Mansfield Hospital, Mansfield, OH, United States; ^8^ Centre for Applied Physics and Radiation Technologies, Sunway University, Sunway, Malaysia; ^9^ School of Mathematics and Physics, University of Surrey, Guildford, United Kingdom; ^10^ Department of Radiation Oncology, University of Toronto, Toronto, ON, Canada; ^11^ Radiation Medicine Program, Princess Margaret Cancer Centre, University Health Network, ON, Canada

**Keywords:** radiotherapy treatment plan, conventional fractionated (CF), hypofractionated (HF), breast cancer, radiobiology model, Halcyon, normal tissue complication probability (NTCP), tumor control probability (TCP)

## Abstract

**Purpose:**

The objective of this research is to compare the efficacy of conventional and hypofractionated radiotherapy treatment plans for breast cancer patients, with a specific focus on the unique features of the Halcyon system.

**Methods and materials:**

The study collected and analyzed dose volume histogram (DVH) data for two groups of treatment plans implemented using the Halcyon system. The first group consisted of 19 patients who received conventional fractionated (CF) treatment with a total dose of 50 Gy in 25 fractions, while the second group comprised 9 patients who received hypofractionated (HF) treatment with a total dose of 42.56 Gy in 16 fractions. The DVH data was used to calculate various parameters, including tumor control probability (TCP), normal tissue complication probability (NTCP), and equivalent uniform dose (EUD), using radiobiological models.

**Results:**

The results indicated that the CF plan resulted in higher TCP but lower NTCP for the lungs compared to the HF plan. The EUD for the HF plan was approximately 49 Gy (114% of its total dose) while that for the CF plan was around 53 Gy (107% of its total dose).

**Conclusions:**

The analysis suggests that while the CF plan is better at controlling tumors, it is not as effective as the HF plan in minimizing side effects. Additionally, it is suggested that there may be an optimal configuration for the HF plan that can provide the same or higher EUD than the CF plan.

## 1 Introduction

Breast cancer is a prevalent form of cancer and one of the leading causes of death among women worldwide. The World Health Organization (WHO) reports that breast cancer is the most commonly diagnosed cancer among women globally, with an estimated 2.3 million new cases in 2020. In the United States, breast cancer accounts for approximately 30% of all new female cancer diagnoses annually, and it is projected that 43,700 women will die from breast cancer in 20232. Despite advancements in diagnosis and treatment, breast cancer remains a significant public health concern. Breast cancer can be effectively treated with radiotherapy, which involves using high-energy radiation to destroy cancer cells and prevent their growth and spread. However, as radiation transmission in matter cannot be precisely controlled, it can affect healthy cells, particularly those near cancer cells. Therefore, an optimal treatment plan should aim to maximize the impact of radiation on the cancerous tissue while minimizing its effects on healthy tissues.

The radiotherapy technique is a critical factor in achieving an effective breast cancer treatment plan. Modern devices, such as the Halcyon system (1), that utilize intensity modulated radiation therapy (IMRT) and volumetric modulated arc therapy (VMAT), provide a simpler way to achieve this compared to less modern devices with fixed radiation beams. The Halcyon offers several advantages over a conventional LINAC in the context of treatment planning. With its single 6 MV flattening filter-free (FFF) X-ray, the Halcyon system delivers a higher dose rate, leading to faster treatment sessions and improved patient comfort. The double-layer multi-leaf collimator (MLC) enables precise beam shaping, enhancing target conformity and sparing surrounding healthy tissues. Additionally, the Halcyon’s faster gantry rotation speed and maximum leaf speed contribute to reduced treatment times, increasing treatment efficiency and minimizing motion-related uncertainties. These combined features make the Halcyon LINAC a particularly choice for treatment planning, ensuring better dose delivery and overall treatment outcomes compared to conventional LINACs. However, given a specific radiotherapy technique, there are various treatment plans that can be constructed, with the total dose and number of treatment fractions being significant parameters. A conventional fractionated (CF) treatment plan for breast cancer typically involves a total dose of 50 Gy administered over 25 treatment fractions. The extended duration of the CF plan limits the capacity to treat a larger number of patients, particularly in developing countries such as Vietnam. Moreover, the cost and travel distance to the radio-therapy center for several weeks can cause financial difficulties for patients. There has been a trend towards using a hypo-fractionated (HF) plan, which has a shorter treatment duration than the CF plan. Although the total dose and number of treatment fractions are lower in an HF plan, the dosage per fraction is higher than that of the CF plan. This trend is evident in the review of Yasemin Bolukbasi and Ugur Selek (2), and studies have confirmed that the HF plan is safe and effective (3–6), yet some inconsistent results exist. For instance, Arezoo Kazemzadeh et al (7) used radiobiological models and found that the tumor control probability and equivalent uniform dose of the CF plan were better than those of the HF plan, contradicting the conclusion of Gloi (8). The review of Youssef and Stanford (9) added that *“breast fibrosis can be a potential side effect of hypofractionated radiotherapy”.* Additionally, there is no standard value for the HF treatment plan, necessitating further research to understand the differences in effectiveness between CF and HF treatment plans. The findings from these studies, as well as the dosimetric data collected, can help to explore more intensified hypofractionated treatment plans, with initial results indicating comparable effectiveness between the plan of 28.5 Gy in 5 fractions and the standard plan of 50 Gy in 25 fractions (10). While the Halcyon system is a state-of-the-art linear accelerator that provides high-quality IMRT and VMAT treatments, there are a limited number of studies on this specific machine. Therefore, the purpose of this study is to evaluate the efficacy of two breast cancer treatment plans based on the Halcyon system using two radiobiological models: the CF plan, which delivers a total dose of 50 Gy over 25 treatment fractions, and the HF plan, which delivers a total dose of 42.56 Gy over 16 treatment fractions.

It is worthy to note that at our hospital, we currently adhere to the National Comprehensive Cancer Network (NCCN) Guideline for treating breast cancer patients (11). In radiation therapy practice, we routinely accept both CF and HF plans according to the NRG Radiation Therapy Oncology Group (RTOG) Template for whole breast photon therapy (12–14). The final decision of which plan should be used in a given case is almost random. This is acceptable because both plans adapt the mandatory requirements of safety, nevertheless we aim to find out which plan.

## 2 Materials and methods

### 2.1 Data collection and manipulation

We collected IMRT treatment plan data for 28 breast cancer patients. Of these patients, 19 chose the CF plan, which consists of a total dose of 50-Gy in 25 fractions, and 9 chose the HF plan, whose total dose and number of treatment fractions are 42.56 Gy and 16, respectively. These IMRT plans were created to be used with the Halcyon system (Varian Medical Systems, Pal Alto, CA). The planning process has been carried out by Medical Physicists using the Eclipse 15.6 (15). All treatment plans considered in this work met the required dose constraints and were approved by physicians at the Hospital.

For each patient, we conducted a computed tomography (CT) scan that encompassed the area from the chin to below the breast crease, extending approximately 6 cm below, with the distance between two successive CT images being 1 mm. The resulting CT images were then input into the Eclipse 15.6 software for contouring and planning. For radiotherapy planning, all contouring of the planning treatment volume (PTV) and organs at risk (OARs), including the cancerous breast, contralateral breast, heart, ipsilateral and contralateral lungs, and spinal cord, was performed manually by a Radiation Oncologist. Contouring data is stored in radiotherapy structure (RT-structure) files, and dose data is stored in radiotherapy dose (RT-dose) files. From these two files, we used the dicompylercore python library (16) to extract the dose volume histogram data for various organs.

### 2.2 Dose volume histogram parameters and radiobiological models

#### 2.2.1 Dosimetric parameters

In the field of radiotherapy modeling, the target volume, such as a tumor, is divided into a large number of voxels. Each voxel may receive a different amount of dose. While it is important to know the dose value of all the voxels to evaluate the treatment plan, storing this information would require a large amount of storage space. Therefore, a more efficient approach is to store the dose volume histogram (DVH), which provides information on the number of voxels that receive a given dose. From DVH data, it is possible to determine the maximum, minimum, and average dose that a voxel in the target volume receives. These parameters are denoted as D_max_, D_min_, and D_mean_.

DVH analysis provides two additional important parameters: D_x%_ and V_RI_. D_x%_ is the minimum dose value received by x% of the total voxels within the target volume, whereas V_RI_ is the relative number of voxels of the target volume that receives the dose of at least RI i.e., the reference isodose. It is noted that for different organs, the values of interest for x% and RI vary. Based on the DVH data and parameters mentioned above, one can deduce specific quantities that can be used to evaluate the effectiveness of a treatment plan. One of them is the homogeneity index (HI), which can be computed as (17)


(1) $$\text{HI}=\frac{{\text{D}}_{2\%}-{\text{D}}_{98\%}}{\text{total dose}}$$

with the total dose being 50 and 42.56 Gy for the CF and HF plans, respectively. Here, D_2%_ and D_98%_ are computed for the cancerous breast, i.e., the PTV. A HI value close to zero means that the dose is homogeneously distributed in the PTV. Another one is the conformity index (CI), which is calculated as


(2) $$\text{CI}=\frac{{\left({\text{V}}_{\text{RI}}\text{ for PTV}\right)}^{2}}{100\times \sum_{\text{all organs}}\left({\text{V}}_{\text{RI}}{\text{ for the i}}^{\text{th}}\text{ organ}\right)}$$

where reference isodose RI = 40.43 and 47.5 Gy for HF and CF plans, respectively. Due to the difference in the definition of some quantities, Eq. 2 in the present work is not the same as the CI formula in the original work (18, 19), yet they are equivalent. In short, the ideal value of CI is 1, i.e., no region outside the PTV receives dose that is equal or more than the reference isodose.

#### 2.2.2 Radiobiological models

In practice, it is difficult to deduce conclusions by comparing the dosimetric parameters of the CF and HF plans because the total doses of these plans are not the same. For instance, the D_min_, D_max_, and D_mean_ of the CF plan are always of greater value than those of the HF plans. Thus, one should use radiobiological models widely applied in the studies of radiotherapy treatment planning (20–22). In this work, we consider three important quantities, equivalent uniform dose (EUD), tumor control probability (TCP), and normal tissue complication probability (NTCP).

First, we determine the EUD based on Niemierko’s phenomenological model (23) as


(3) $$EUD={\left(\sum_{total\;volume}\vartheta_{i}EQD_{i}^{a}\right)}^{1/a}$$

where $\vartheta_{i}$ is the number of voxels that receive a dose of ${\text{EQD}}_{\text{i}}$ and a is a model parameter that is specific to the organ considered. The EQD denotes the biologically equivalent physical dose of 2 Gy and is computed as


(4) $$EQD=D\times \frac{\frac{\alpha }{\beta }+\frac{D}{n_{f}}}{\frac{\beta }{\alpha }}$$

with $\frac{\alpha }{\beta }$ , D, and $n_{f}$ being the tissue-specific linear-quadratic parameter of the organ being exposed, the total dose, and the number of fractions of the treatment plan, respectively.

For the TCP, we apply the Poisson linear quadratic (PoissonLQ) radiobiological model (24), namely


(5) $$\text{TCP}\left(\text{D}\right)=\prod_{i}^{M}{\left[\text{exp}\left(-\text{exp}\left[e\gamma -\frac{EQD_{i}}{D_{50}}\left(e\gamma -\ln\left(\ln\left(2\right)\right)\right)\right]\right)\right]}^{\frac{v_{i}}{v_{\text{ref}}}}$$

here, e is the Euler’s number, $\frac{v_{i}}{v_{\text{ref}}}$ is the relative volume of voxel i compared to the references volume, i.e., the total volume of the organ considered, and M is the total number of voxels. Whereas γ and $D_{50}$ are the maximum normalized gradient of the dose response curve and dose giving a 50% response probability of the organ of interest, respectively.

For the NTCP, Lyman-Kutcher-Burman (LKB) model (24) is used. This model defines the NTCP as


(6) $$\text{NTCP}\left(\text{D}\right)=\frac{1}{\sqrt{2\text{π}}}\int_{\infty }^{t}e^{\frac{-x^{2}}{2}}dx$$

with


(7) $$\text{t}=\frac{{\text{D}}_{\text{eff}}-{\text{D}}_{50}}{\text{m}.{\text{D}}_{50}}$$

and


(8) $${\text{D}}_{\text{eff}}={\sum_{i=1}^{M}\left(\frac{v_{i}}{v_{\text{ref}}}{\text{EQD}}_{i}^{1/\text{n}}\right)}^{n}$$

Within Eqs. 6, 7, and 8, *m* and *n* are the slope of the response curve and parameter reflecting the biological properties of the organ specifying volume dependence. TCP and NTCP are probability, thus their possible outcome of TCP and NTCP ranges from 0 to 1. The objective of a treatment plan is to simultaneously maximize TCP and EUD and minimize NTCP.

The radiobiology models described above are employed in the pyRadioBiology python package contributors (25), thus it is used to perform the calculation of TCP, NTCP, and EUD in the present work. The values of the parameters and their corresponding references are summarized in **Table 1**.

**Table 1:** Values of the parameters used within radiobiology model calculations.

Parameter	Organ	Value	Value References
$\frac{\alpha }{\beta }$ (unitless)	Breast (PTV), heart, and lung	4	Owen et al., 2006 (3)
*a* (unitless)	Breast (PTV)	-7.2	Okunieff et al., 1995; Horton et al., 2006 (26, 27)
*γ* (unitless)	Breast (PTV)	1.3	Okunieff et al., 1995; Horton et al., 2006 (26, 27)
*D*_50_ (Gy)	Breast (PTV)	30.89	Okunieff et al., 1995; Horton et al., 2006 (26, 27)
*D*_50_ (Gy)	Heart	48	Luxton et al., 2007; Oinam et al., 2011 (28, 29)
*D*_50_ (Gy)	Lung	37.6	Oinam et al., 2011; Semenenko VA, 2008 (29, 30)
*M (*unitless)	Heart	0.1	Luxton et al., 2007; Oinam et al., 2011 (28, 29)
*n* (unitless)	Heart	0.35	Luxton et al., 2007; Oinam et al., 2011 (28, 29)
*M* (unitless)	Lung	0.35	Oinam et al., 2011; Semenenko et al., 2008 (29, 30)
*n* (unitless)	Lung	0.87	Oinam et al., 2011; Semenenko et al., 2008 (29, 30)

### 2.3 Plan evaluation

All the dosimetric parameters, including the CI and HI, and radiobiology model quantities presented in section 2.2 are determined for each patient. It is noted that the EUD and TCP are calculated for the PTV, namely the cancerous breast, whereas the NTCP is calculated for the heart and lung (ipsilateral and contralateral parts). The latter are important normal tissues that are in the vicinity of the PTV and thus need to be monitored.

To compare the effectiveness of the CF and HF plans, we compared CI, HI, EUD, TCP, and NTCP corresponding to these two groups of patients using the independent sample *t*-test. The significant level was considered at a *p*-value of 0.05, corresponding to the reliability of 95%.

In short, the ideal treatment plan should have:

- CI equal to 1,
- HI equal to 0,
- TCP equal to 1,
- NTCP equal to 0,
- and EUD is as high as possible.

## 3 Results and discussion

**Figure 1:**
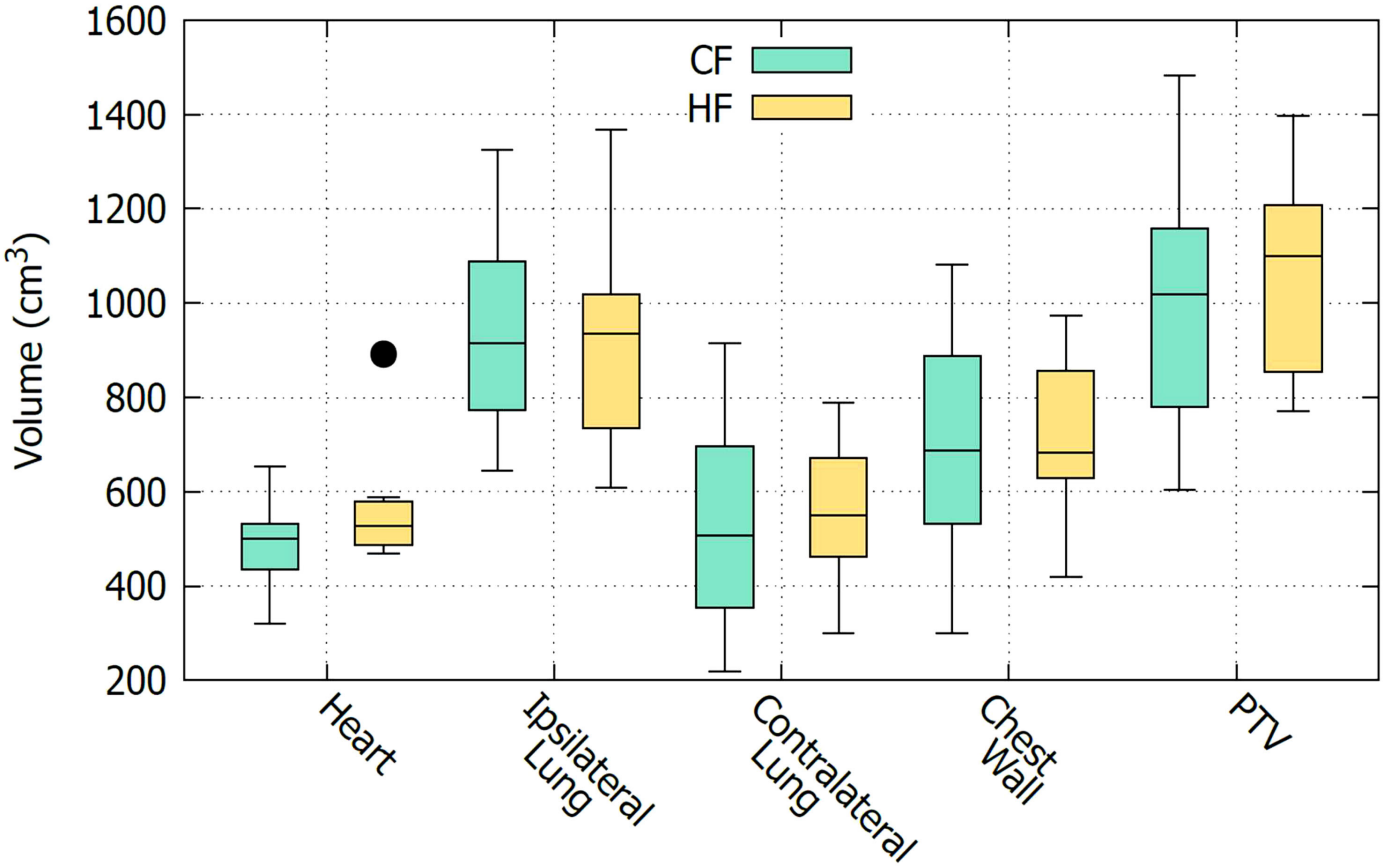
Comparison of the sizes of organs between two groups of patients.

As described in section 2.1, CF and HF plans are applied to two separate groups of patients. To compare the effectiveness of the CF and HF plans, it is important to ensure that the two groups of patients have similar characteristics. However, in this study which only considered DVH data, it is only necessary to ensure that the organs being treated are anatomically comparable between the two groups. Other demographic factors, such as age and gender, do not affect the analysis. **Figure 1** shows a boxplot to visually compare the size of organs of the two groups of patients. It is seen that the sizes of the organs of these two groups are comparable. This observation is confirmed with the *t*-test comparison given in **Table 2**. It is displayed that all the *t*-values are nearly zero except for the heart. The high *t*-value corresponding to the heart might be due to the bias of an outlier data point as can be seen in **Figure 1**. In fact, it may be preferable in a planning study to simulate both treatments in each patient, as this enables a paired comparison and even a voxel-by-voxel comparison of physical dose, and thus eliminates the need to determine whether the groups are comparable. However, a study based on actually delivered plans, such as the one presented here, has its own advantage in that it provides insight into the practical clinical experience, including the occurrence of any adverse effects. For this reason, a similar strategy was also carried out by many other works, see e.g., Refs (5, 7, 8, 21).

**Table 2:** Comparison of the size of organs of the CF and HF groups of patients using independent sample *t*-test.

Organ	CF (cm^3^)	HF (cm^3^)	*t*−value	*p*−value
Heart	490 ± 79	569 ± 129	−2.01	0.054
Ipsilateral Lung	928 ± 192	931 ± 234	−0.04	0.967
Contralateral Lung	1000 ± 268	1080 ± 220	−0.77	0.449
Chest Wall	515 ± 187	541 ± 164	−0.36	0.725
PTV	673 ± 206	706 ± 184	−0.40	0.691

Values corresponding to the CF and HF plans are presented as mean ± standard deviation.

**Table 3** presents a comparison of the CI, HI, EUD, TCP, and NTCP values of CF and HF plans using an independent sample t-test. The results show that both treatment plans have a high CI value (>0.95), indicating that they effectively concentrate radiation dose within the PTV, i.e., the cancerous breast, while minimizing exposure to healthy tissues. This is an important aspect of radiation therapy, as it reduces the likelihood of side effects and complications. The results suggest that both treatment plans are well-suited for treating breast cancer patients using the Halcyon system. A comparison with prior research demonstrates that the CI value is independent of the treatment plan, Refs (9, 10) also revealing nearly identical CI values for the CF and HF treatment plans. Indeed, a high value of CI can be attained through enhanced therapy methods. For example, the CF plan combined with the IMRT and VMAT treatment techniques in our work yields a CI value of 0.96, whereas the same treatment plan with the 3D-CRT technique in the work of Kazemzadeh A et al. (9) yields only 0.89.

**Table 3:** Comparison of the CI, HI, EUD, TCP, NTCP of CF and HF plans using independent sample t-test.

	CF	HF	*t*−value	*p*−value
CI	0.96 ± 0.01	0.95 ± 0.02	0.33	0.743
HI	0.08 ± 0.01	0.10 ± 0.02	−4.40	#
EUD (PTV)	52.39 ± 0.23	49.14 ± 1.27	11.00	#
TCP (PTV)	95.5 ± 0.1%	93.6 ± 0.3%	23.67	#
NTCP (Heart)	#	#	−0.76	0.453
NTCP (Ipsilateral Lung)	4.9 ± 0.6%	3.7 ± 0.3%	6.14	#
NTCP (Contralateral Lung)	0.38 ± # %	0.34 ± 0.04%	2.52	0.018

The symbol # indicates that the value is nearly equal to zero.

Values corresponding to the CF and HF plans are presented as mean ± standard deviation.

When the HI values are compared, the CF plan does a little better than the HF plan. This means that the CF plan was able to spread the radiation dose more evenly across the PTV than the HF plan. A uniform distribution of radiation dose is essential, as an uneven dose distribution may cause certain parts of the cancerous organ to receive excessive radiation, while others receive insufficient radiation to effectively destroy the cancerous cells. This can result in incomplete treatment, allowing cancerous cells to survive and recover after the therapy. Therefore, a homogeneous distribution of radiation dose within the PTV is desirable in order to achieve optimal therapeutic outcomes. In general, the HI values of both the CF and HF plans in the present work are fairly good, as they are less than 0.10, indicating that the maximum discrepancy of the dose received at two random locations inside the PTV is less than 10%.

The TCP value of the CF plan, which is 96.0 ± 0.1%, is better than that of the HF, which is 94.0 ± 0.3%, as expressed by the large *t*-value of 23.67 together with a *p*-value of almost zero. This result for the TCP value is consistent with that reported in Ref (9). Yet inconsistent with that given in Ref (10), in which the TCP value of the CF plan is lower than the HF plan. We should note that the authors of Ref (10) reported their TCP values with extremely large uncertainty, in contrast to the small uncertainty reported in this study and Ref (9).

The NTCP values for the heart of both CF and HF plans are all extremely close to zero. For the lung, the NTCP values of the HF plan are better than those of the CF. In addition, the NTCP of the ipsilateral lung is 12 times larger than that of the contralateral lung for both treatment plans. It is evident because the ipsilateral lung is located on the same side as the PTV, so it receives a higher dose than the contralateral lung. In general, the CF plan is more effective at controlling tumor growth, as suggested by its better TCP values, yet the HF plan causes fewer side effects and complications for the patients, demonstrated by its better NTCP values, particularly the lung. We should note that this claim is only valid for treatments utilizing IMRT techniques on a Halcyon system, also noting that in the study of Kazemzadeh A et al. (9), in which the treatment were made on an ARTISTE radiation therapy system (SIEMENS) using 3D-CRT technique, the NTCP for the lung of the HF plan was reported to be worse than that of the CF.

For the EUD values, that of the CF plan is higher than that of the HF. This is expected because the total dose of the CF plan is greater than that of the HF case. However, there is an interesting observation that the relative ratio between the EUD and the total dose in the CF plan is lower than in the HF plan. In the CF plan, a total dose of 50 Gy results in a EUD of 52.39 Gy (107%), whereas a 42.56 Gy total dose in the HF plan causes a EUD of 49.14 (114%). A similar effect is seen when comparing V_RI_ of the CF and HF plans for the PTV and chest wall, as shown in **Table 4**. With the same relative reference isodose of 105% with respect to the total dose (i.e., 53.5 Gy versus 50 Gy and 45.53 Gy versus 42.56 Gy), the V_RI_ value of the CF plan is greater than that of the HF plan, whereas the V_RI_ values with the relative reference isodoses of 95% and 100% show the contrary. This observation suggests that the HF plan delivers doses to the PTV more effectively than the CF plan. It also implies that the current configuration of the HF plan may not be optimal, and there may exist a treatment plan with a higher EUD, even greater than the EUD obtained from the CF plan. In addition, behaviour of the EUD observed in our work was not shown in Ref (9). Since both works considered the same CF and HF treatment plans but used different treatment techniques, namely IMRT and VMAT in this work versus 3D-CRT in Ref (7), it is possible that the choice of treatment plan varies with the treatment technique.

**Table 4:** Comparison between V_53.5_ and V_45.53_, V_50_ and V_42.56_, and V_47.5_ and V_40.43_ of the CF and HF plans, for the PTV and chest wall.

Organ	Parameters	CF	HF	*t*−value	*p*−value
PTV	V_95%_	98.51 ± 1.33	93.69 ± 3.80	3.80	#
	V_100%_	99.96 ± 0.08	99.56 ± 0.08	3.10	0.005
	V_107%_	7.14 ± 4.62	11.00 ± 4.28	-2.11	0.045
Chest Wall	V_95%_	99.96 ± 0.07	99.50 ± 0.70	2.91	0.007
	V_100%_	98.48 ± 1.44	93.41 ± 4.30	4.70	#
	V_107%_	8.16 ± 4.99	12.25 ± 3.99	2.15	0.041

The values of 47.5, 50, and 53.5 correspond to 95%, 100%, and 107% of the total dose of the CF plan, whereas those of 40.43, 42.56, and 45.53 correspond to 95%, 100%, and 107% of the total dose of the HF plan.

Values corresponding to the CF and HF plans are presented as mean ± standard deviation.

The symbol # indicates that the value is almost equal to zero.

Overall, the results of the present work suggest that, for the Halcyon system with IMRT technique, the CF plan is better at controlling tumor growth than the HF plan, but the HF plan is better at keeping side effects and complications to a minimum. The HF plan has the additional advantage of a shorter treatment duration. In addition, the analysis of the EUD shows that there might exist a configuration of the HF plan that can provide the same or even a greater EUD value than that of the CF plan. In future studies, we intend to perform a search to find the optimal configuration of the HF plan. Moreover, although all the comparisons in this work are based on a *t-*test with a replication probability of more than 95%, recalling that we accept the hypothesis only if the *p*-value is less than 0.05. Further, it is to be acknowledged that the number of samples, 28 patients, is quite small. Therefore, the obtained results must be considered with caution until further studies with a larger sample size are conducted.

## 4 Conclusions

In conclusion, this study compared the effectiveness of conventional and hypofractionated radiotherapy treatment plans for breast cancer patients using radiobiological models and analyzed dosimetric parameters. The results suggest that while the CF plan is more effective in tumor control, the HF plan is better at minimizing side effects and complications and has the advantage of a shorter treatment duration. Additionally, the analysis of the EUD suggests that there might be an optimal configuration of the HF plan that can provide the same or even a higher EUD value than that of the CF plan.

## Data availability statement

The raw data supporting the conclusions of this article will be made available by the authors, without undue reservation.

## Author contributions

DT: Conceptualization, Data curation, Methodology, Project administration, Software, Supervision, Validation, Writing – original draft, Writing – review & editing. LT: Conceptualization, Data curation, Methodology, Software, Writing – original draft. NN: Conceptualization, Methodology, Software, Writing – original draft. HS: Data curation, Writing – original draft. TL: Data curation, Writing – original draft. XN: Conceptualization, Investigation, Writing – original draft, Writing – review & editing. PS: Conceptualization, Writing – original draft, Writing – review & editing. DB: Investigation, Supervision, Writing – review & editing. JC: Conceptualization, Investigation, Project administration, Writing – original draft, Writing – review & editing.

## References

[1] Varian. Halcyon treatment delivery system (2023). Available at: https://www.varian.com/products/radiotherapy/treatment-delivery/halcyon (Accessed April 9, 2023).

[2] Bolukbasi Y Selek U. Modern radiotherapy era in breast cancer, in: Breast cancer - from biology to medicine. Available at: https://www.intechopen.com/chapters/53531 (Accessed April 9, 2023).

[3] Owen JR Ashton A Bliss JM Homewood J Harper C Hanson J. Effect of radiotherapy fraction size on tumour control in patients with early-stage breast cancer after local tumour excision: long-term results of a randomised trial. Lancet Oncol (2006) 7(6):467–71. doi: 10.1016/S1470-2045(06)70699-4 16750496

[4] Whelan TJ Pignol JP Levine MN Julian JA MacKenzie R Parpia S. Long-term results of hypofractionated radiation therapy for breast cancer. N Engl J Med (2010) 362(6):513–20. doi: 10.1056/NEJMoa0906260 20147717

[5] Abram WP Clarke J McAleer JJ Graham JD Riddle P Goodman S. The UK Standardisation of Breast Radiotherapy (START) Trial A of radiotherapy hypofractionation for treatment of early breast cancer: a randomised trial. Lancet Oncol (2008) 9(4):331–41. doi: 10.1016/S1470-2045(08)70077-9 PMC2323709 18356109

[6] Haviland JS Owen JR Dewar JA Agrawal RK Barrett J Barrett-Lee PJ. The UK Standardisation of Breast Radiotherapy (START) trials of radiotherapy hypofractionation for treatment of early breast cancer: 10-year follow-up results of two randomised controlled trials. Lancet Oncol (2013) 14(11):1086–94. doi: 10.1016/S1470-2045(13)70386-3 24055415

[7] Kazemzadeh A Abedi I Amouheidari A Shirvany A. A radiobiological comparison of hypo-fractionation versus conventional fractionation for breast cancer 3D-conformal radiation therapy. Rep Pract Oncol Radiother. (2021) 26(1):86–92. doi: 10.5603/RPOR.a2021.0015 34046218 PMC8149130

[8] Gloi AM. A broad evaluation of left breast radiotherapy. Am J Biomed Sci (2019) 152:152–71. doi: 10.5099/aj190300153

[9] Youssef A Stanford J. Hypofractionation radiotherapy vs. Conventional fractionation for breast cancer: A comparative review of toxicity. Cureus. (2018) 10(10):e3516. doi: 10.7759/cureus.3516 30648051 PMC6318139

[10] FAST Trialists group Agrawal RK Alhasso A. First results of the randomised UK FAST Trial of radiotherapy hypofractionation for treatment of early breast cancer (CRUKE/04/015). Radiother Oncol (2011) 100(1):93–100. doi: 10.1016/j.radonc.2011.06.026 21752481

[11] Goetz MP Gradishar WJ Anderson BO Abraham J Aft R Allison KH. NCCN guidelines insights: Breast cancer, version 3.2018: Featured updates to the NCCN guidelines. J Natl Compr Cancer Network. (2019) 17(2):118–26. doi: 10.6004/jnccn.2019.0009 30787125

[12] Kainz K Huang Mi Xiao Y Li XA Moran. JM. NRG protocol radiation therapy template. Available at: https://www.nrgoncology.org/ciro-breast (Accessed August 25, 2023).

[13] Freedman GM White JR Arthur DW Allen Li X Vicini FA. Accelerated fractionation with a concurrent boost for early stage breast cancer. Radiotherapy Oncol (2013) 106(1):15–20. doi: 10.1016/j.radonc.2012.12.001 23333014

[14] Vicini FA Winter K Freedman GM Arthur DW Hayman JA Rosenstein BS. NRG RTOG 1005: A phase III trial of hypo fractionated whole breast irradiation with concurrent boost vs. Conventional whole breast irradiation plus sequential boost following lumpectomy for high risk early-stage breast cancer. Int J Radiat Oncology Biology Physics. (2022) 114(3):S1. doi: 10.1016/j.ijrobp.2022.07.2320

[15] Eclipse. Eclipse treatment planning system, in: (2023). Available at: https://www.varian.com/products/radiotherapy/treatment-planning/eclipse (Accessed April 9, 2023).

[16] Panchal A Couture G Galler N Hall DC Wakita A. dicompyler/dicompyler-core v0.5.5. Zenodo. doi: 10.5281/zenodo.3236628

[17] Wu Q Mohan R Morris M Lauve A Schmidt-Ullrich R. Simultaneous integrated boost intensity-modulated radiotherapy for locally advanced head-and-neck squamous cell carcinomas. I: dosimetric results. Int J Radiat Oncol Biol Phys (2003) 56(2):573–85. doi: 10.1016/s0360-3016(02)04617-5 12738335

[18] Shaw E Kline R Gillin M Souhami L Hirschfeld A Dinapoli R. Radiation Therapy Oncology Group: radiosurgery quality assurance guidelines. Int J Radiat Oncol Biol Phys (1993) 27(5):1231–9. doi: 10.1016/0360-3016(93)90548-a 8262852

[19] Wambersie A. ICRU report 62, prescribing, recording and reporting photon beam therapy (Supplement to ICRU 50) – ICRU. Available at: https://www.icru.org/report/prescribing-recording-and-reporting-photon-beam-therapy-report-62/ (Accessed April 9, 2023).

[20] Chow JCL Jiang R. Dose-volume and radiobiological dependence on the calculation grid size in prostate VMAT planning. Med Dosim. (2018) 43(4):383–9. doi: 10.1016/j.meddos.2017.12.002 29373184

[21] Chow JCL Jiang R Xu L. Dosimetric and radiobiological comparison of prostate VMAT plans optimized using the photon and progressive resolution algorithm. Med Dosim. (2020) 45(1):14–8. doi: 10.1016/j.meddos.2019.04.004 31103251

[22] Tai DT Oanh LT Phuong PH Sulieman A Abolaban FA Omer H. Dosimetric and radiobiological comparison in head-and-neck radiotherapy using JO-IMRT and 3D-CRT. Saudi J Biol Sci (2022) 29(8):103336. doi: 10.1016/j.sjbs.2022.103336 35754762 PMC9213241

[23] Niemierko A. Reporting and analyzing dose distributions: a concept of equivalent uniform dose. Med Phys (1997) 24(1):103–10. doi: 10.1118/1.598063 9029544

[24] Laboratories R. RayStation 6 reference manual, raySearch laboratories. (2017).

[25] Sachpazidis I. pyradiobiology: pyradiobiology is a package for radiobiological modeling (TCP, NTCP, EUD, gEUD) with Python. Available at: https://www.sachpazidis.com/pyRadioBiology/ (Accessed April 9, 2023).

[26] Okunieff P Morgan D Niemierko A Suit HD. Radiation dose-response of human tumors. Int J Radiat Oncol Biol Phys (1995) 32(4):1227–37. doi: 10.1016/0360-3016(94)00475-z 7607946

[27] Horton JK Halle JS Chang SX Sartor CI. Comparison of three concomitant boost techniques for early-stage breast cancer. Int J Radiat OncologyBiologyPhysics. (2006) 64(1):168–75. doi: 10.1016/j.ijrobp.2005.07.004 16198507

[28] Luxton G Keall PJ King CR. A new formula for normal tissue complication probability (NTCP) as a function of equivalent uniform dose (EUD). Phys Med Biol (2008) 53(1):23–36. doi: 10.1088/0031-9155/53/1/002 18182685

[29] Oinam AS Singh L Shukla A Ghoshal S Kapoor R Sharma SC. Dose volume histogram analysis and comparison of different radiobiological models using in-house developed software. J Med Phys (2011) 36(4):220–9. doi: 10.4103/0971-6203.89971 PMC3249733 22228931

[30] Semenenko VA Li XA. Lyman-Kutcher-Burman NTCP model parameters for radiation pneumonitis and xerostomia based on combined analysis of published clinical data. Phys Med Biol (2008) 53(3):737–55. doi: 10.1088/0031-9155/53/3/014 18199912

